# Intraoperative colon perfusion assessment using multispectral imaging

**DOI:** 10.1364/BOE.435118

**Published:** 2021-11-12

**Authors:** Neil T. Clancy, António S. Soares, Sophia Bano, Laurence B. Lovat, Manish Chand, Danail Stoyanov

**Affiliations:** 1 Wellcome/EPSRC Centre for Interventional and Surgical Sciences, University College London, UK; 2 Department of Medical Physics and Biomedical Engineering, University College London, UK; 3 Division of Surgery and Interventional Sciences, University College London, UK; 4 Department of Computer Science, University College London, UK

## Abstract

In colorectal surgery an anastomosis performed using poorly-perfused, ischaemic bowel segments may result in a leak and consequent morbidity. Traditional measures of perfusion assessment rely on clinical judgement and are mainly subjective, based on tissue appearance, leading to variability between clinicians. This paper describes a multispectral imaging (MSI) laparoscope that can derive quantitative measures of tissue oxygen saturation (*SO_2_*). The system uses a xenon surgical light source and fast filter wheel camera to capture eight narrow waveband images across the visible range in approximately 0.3 s. Spectral validation measurements were performed by imaging standardised colour tiles and comparing reflectance with ground truth spectrometer data. Tissue spectra were decomposed into individual contributions from haemoglobin, adipose tissue and scattering, using a previously-developed regression approach. Initial clinical results from seven patients undergoing colorectal surgery are presented and used to characterise measurement stability and reproducibility in vivo. Strategies to improve signal-to-noise ratio and correct for motion are described. Images of healthy bowel tissue (in vivo) indicate that baseline *SO_2_* is approximately 75 ± 6%. The *SO_2_* profile along a bowel segment following ligation of the inferior mesenteric artery (IMA) shows a decrease from the proximal to distal end. In the clinical cases shown, imaging results concurred with clinical judgements of the location of well-perfused tissue. Adipose tissue, visibly yellow in the RGB images, is shown to surround the mesentery and cover some of the serosa. *SO_2_* in this tissue is consistently high, with mean value of 90%. These results show that MSI is a potential intraoperative guidance tool for assessment of perfusion. Mapping of *SO_2_* in the colon could be used by surgeons to guide choice of transection points and ensure that well-perfused tissue is used to form an anastomosis. The observation of high mesenteric *SO_2_* agrees with work in the literature and warrants further exploration. Larger studies incorporating with a wider cohort of clinicians will help to provide retrospective evidence of how this imaging technique may be able to reduce inter-operator variability.

## 1. Introduction

Tissue perfusion is typically assessed intraoperatively using visual cues, such as colour, motility and surface changes, describing the appearance of the tissue. This judgement is critical to avoid surgical complications relating to ischaemia. In colorectal surgery, for example, excision of diseased colon often necessitates an anastomosis, whereby two sections of bowel are joined together with sutures or staples. The key determinant to avoid anastomotic failure is a good blood supply to the bowel being anastomosed. Failure rates in some types of bowel surgery can be as high as 12-19% [1,2], particularly those lower in the pelvis. This has serious consequences for the patient, associated with significant morbidity, and requires urgent and intensive intervention. Therefore, the surgeon must try to determine the optimal position for an anastomosis where there is a good perfusion. Traditionally, this is a subjective clinical assessment. Previous point probe studies of tissue oxygenation have indicated that low perianastomotic oxygenation is associated with subsequent complications [3], whereas a higher intraoperative *SO_2_* has been observed as a feature of successful anastomoses [4]. Therefore, an objective, quantitative method to assess aspects of tissue health such as oxygen saturation could help to guide surgeons during the procedure, lead to greater consistency in anastomosis formation, and reduce the incidence of post-operative complications.

Optical imaging methods have been explored as a means to objectively quantify perfusion in an organ non-invasively. Fluorescence image-guided surgery (FIGS) is a technique that increases the contrast between perfused and non-perfused tissue using an exogenous fluorescent dye (indocyanine green [ICG]) [5,6]. However, injection of the fluorophore is necessary, and the technique does not provide oxygenation information. Laser Doppler [7] and laser speckle [8] contrast imaging detect differences in backscattered laser light between static and moving cells to estimate blood flow and, with multiple wavelength laser sources, oxygenation measurements are possible. These systems are sensitive to gross tissue motion, and have a high degree of complexity.

Multispectral imaging (MSI) is an optical technique that measures wavelength-dependent changes in the amount of light reflected by tissue and uses them to infer the underlying physical properties, including oxygen saturation [9]. In the visible wavelength range optical absorption in abdominal organs is dominated by oxygenated and deoxygenated haemoglobin. Fat and bile, although accounting for a much lower overall volume fraction, may also contribute significantly in certain locations such as mesenteric adipose tissue and the gall bladder, respectively. The technique has been demonstrated in multiple organs and surgical applications including kidney [10], bowel [11,12], liver [13], brain [14], breast [15] and head-and-neck [16,17]. MSI has also been validated as a perfusion assessment tool in bowel imaging [11] and applied in animal models of uterine transplantation [18]. Although spectral scanning may be performed on the illumination side [19], we prefer implementation at the detector due to greater out-of-band signal rejection and compatibility with existing clinical hardware [9].

In this paper we present a laparoscopic MSI system that uses a filter wheel-based spectral camera to offer fast datacube collection (∼3 image stacks per second), efficient light throughput and high spatial resolution (1.5 MP), which enables accurate collection of spectral data. Results from a clinical pilot study to track *SO_2_* as a measure of organ perfusion demonstrate the first human data from the system during colorectal surgery on imaging the bowel. The device is described and characterised before an analysis of the surgical images obtained.

## 2. Methods

### 2.1 Multispectral imaging laparoscope

**Fig. 1.**
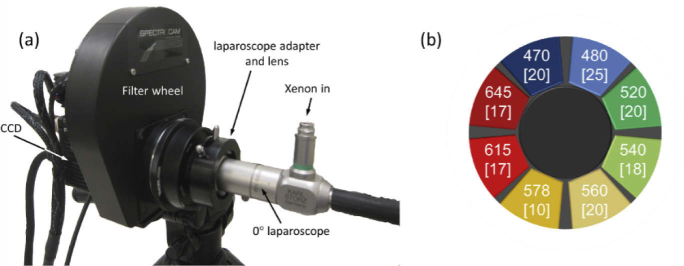
(a) Photograph of the MSI system set-up used in the clinic. Illumination was provided by a xenon lamp connected to the laparoscope using a fibre optic light cable. Reflected light was captured by the laparoscope optics and focussed onto the CCD using an imaging lens housed in the adapter. The camera was mounted on a tripod that could be easily moved to the bedside during measurements, while the light source and computer were carried on a stainless steel trolley. (b) Schematic of the filter wheel configuration with centre wavelengths (in nm) indicated. The numbers in parentheses refer to the full-width at half-maximum (FWHM) of each filter’s spectral transmission profile.

The MSI laparoscope is shown in Fig. 1(a) and consists of a 0° laparoscope (KARL STORZ, GmbH, Germany), 40 mm, 1” diameter, achromatic imaging lens, and a fast filter wheel multispectral imaging camera (SpectroCam; Ocean Insight, USA). The laparoscope is fixed focus, optimised at 10 cm, with depth of field extending to approximately 5 cm and >20 cm. Illumination is provided by a xenon light source (XL300; Lemke, Germany) delivered to the laparoscope via a fibre optic light cable (KARL STORZ, GmbH, Germany). The SpectroCam has a 1.5 MP 12 bit camera sensor that runs at 30 fps, synchronised to the spinning filter wheel. That means that a stack of images, one corresponding to each of the eight interchangeable filters, can be acquired in approximately 0.3 s.

**Fig. 2.**
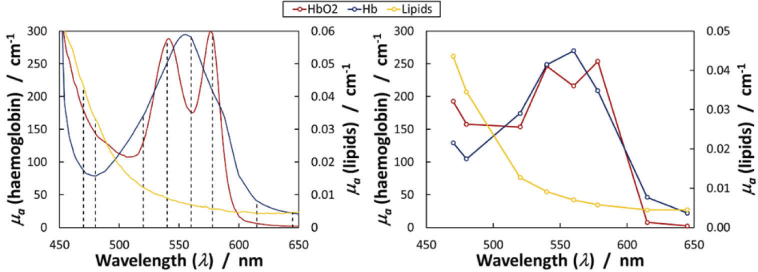
Spectral characteristics of the MSI system. (a) Absorbance properties of expected major chromophores in surgically-exposed tissue obtained from widely-used sources [20]. The centre wavelengths of the SpectroCam filters are indicated by the dashed lines. (b) The pure component spectra measured by the camera are convolutions of the spectra shown in (a) with the spectral shape of each filter’s passband.

The filters, chosen from available stock, were picked to maximise sensitivity to the difference spectrum of oxygenated and deoxygenation haemoglobin. This serves to optimise the dynamic range of the instrument for tissue oxygenation imaging. The filters have a Gaussian transmission profile and their bandwidth, expressed as the full-width at half-maximum (FWHM; Fig. 1(b)), was designed to strike a balance between optical throughput and spectral resolution. The known absorbance spectra of the main chromophores present in the imaged bowel tissue (Fig. 2(a)) is interpreted by the camera as a convolution with the bandwidth of each filter (Fig. 2(b)). A ‘dark spectrum’ (as described in [11]) with xenon switched off was recorded at the start of an acquisition and subtracted from the measured signal to correct for any potential offset effect. A white reflectance standard Labsphere, Inc., USA) was used to correct for the spectral sensitivity of the system and convert reflected intensity to absorbance data [11].

### 2.2 Tissue property estimation

This system uses a linear regression model employed in previous studies [11,21–23] that makes the following assumptions and approximations: (i) the only optical absorbers in the field-of-view are oxyhaemoglobin (*HbO_2_*), deoxyhaemoglobin (*Hb*) and adipose tissue (lipids), (ii) scattering is independent of wavelength (*λ*), (iii) the pathlength travelled by each photon is equal. This allows the absorbance (*A*) to be expressed, using the modified Beer-Lambert law (Eq. (1)), as a sum of the contributions of chromophores and light lost due to scattering:

(1) $$A(\lambda )=[HbO_{2}]\varepsilon_{HbO_{2}}(\lambda )+[Hb]\varepsilon_{Hb}(\lambda )+[Fat]\varepsilon_{Fat}(\lambda )+G$$

where *ε* is the molar extinction coefficient, *G* is an offset to account for scattering, and the terms in square brackets represent relative concentrations. The quality-of-fit is quantified using the coefficient of determination (*CoD*) or r^2^ value, which describes the fraction of the variance in the data explained by the model. These calculations are performed for every spatial pixel location in the image, resulting in the creation of concentration maps corresponding to each of the free variables in Eq. (1) as well as a *CoD* map. These are then used to generate maps of total haemoglobin (*THb* = *HbO_2_ *+* Hb*) and *SO_2_* (*HbO_2_*/*THb*). Pixels with *CoD* less than 0.9 or a negative concentration value are assigned a ‘0’ value and are excluded from quantitative analyses. An equivalent RGB image is generated from the MSI datacube by summing the eight wavebands, weighted by the known RGB filter response of a colour camera, to generate the red, green and blue colour planes. The *SO_2_* result is overlaid on the RGB image with transparency weighted by *THb* [11].

### 2.3 Validation experiments

**Fig. 3.**
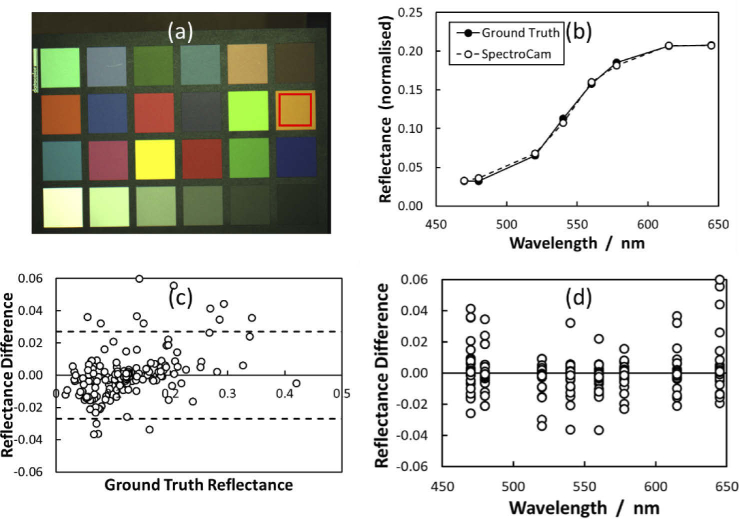
Spectral reflectance validation. (a) A standardised colour checker card (SpyderCheckr) was imaged and the mean spectral reflectance within an ROI in each panel was calculated. (b) The MSI reflectance values were compared with the ground truth, measured using a high-resolution spectrometer, example shown here corresponds to red region in (a). (c) Bland-Altman plot showing the difference in reflectance measurements between SpectroCam and the ground truth. (d) Reflectance difference plotted as a function of wavelength.

Validation of the spectral imaging capabilities of the system was accomplished by imaging a standardised colour checker card (SpyderCheckr) shown in Fig. 3 (a). Ground truth reflectance of each panel was obtained using a high-resolution spectrometer (Thorlabs). The ground truth data was convolved with the transmission spectrum of each filter, in a similar manner to the haemoglobin data described in Section 1, to make this comparable to the MSI imaging results. The card was imaged with the MSI system and the mean reflectance values from each panel were calculated and plotted against the centre wavelength of each passband.

This technique has previously been validated *in vivo* in animal experiments where it was used to follow ischaemia in porcine small bowel following ligation of the mesenteric arcade [11]. These previous studies used a liquid crystal variable filter (LCTF) to generate the spectral datacube. Although offering flexibility in wavelength selection this suffered from poor light throughput, which necessitated long camera integration times (>100 ms) particularly in the blue spectral region. The consequently long datacube acquisition time (up to 7 s) meant that the system was vulnerable to significant motion artefacts due to peristalsis and breathing. The system presented in this study improves on these limitations by using high throughput filters (>90% transmission) to achieve short integration times (33 ms). Along with an optimised wavelength selection (8 bands) [24] the total datacube acquisition time is reduced to 0.3 s, decreasing the risk of significant motion-induced errors.

### 2.4 Clinical measurements

Seven patients attending hospital for colorectal resection were asked to participate in the study and provided written informed consent for video data collection during surgery according to institutional guidelines and approval. There was no change to standard clinical care during this study. The imaging system was brought into the theatre at the beginning of the operation and kept to one side of the room. After resection of the diseased segment of colon, the free end was brought outside the abdomen for inspection of the perfusion. The surgeons made their choice of transection point and marked the position by laying a forceps beside the location. The MSI system was brought to the bedside and positioned so that the segment to be measured was centred and approximately filled the field-of-view. This corresponded to a working distance of approximately 10 cm. Main theatre lights were switched off while MSI acquisition took place. Multiple acquisitions of MSI datacubes were acquired over a period of 20 s. Camera exposure time was set to its maximum value (33 ms) and the gain setting was chosen to maximise signal-to-noise ratio. Once data acquisition was complete the main theatre lights were switched on and the MSI system moved back from the bedside, allowing the surgery to recommence. The total time taken to acquire MSI data for each patient was less than five minutes out of a typical total procedure time of ∼1.5 hours. All image and data processing was completed postoperatively and therefore the surgeons were not provided with any MSI information.

## 3. Results

### 3.1 Spectral validation

The results of the spectral validation experiment are shown in Fig. 3. The mean spectra recorded by SpectroCam are a close match to the ground truth, with the Bland-Altman analysis showing a mean measurement difference between the devices of zero, with 95% of the variability within ±0.027. When differences are plotted as a function of wavelength (Fig. 3(d)) it can also be seen that variance in the errors is approximately constant across the spectrum, with marginally higher deviations at 470 and 645 nm.

### 3.2 Clinical measurements

**Fig. 4.**
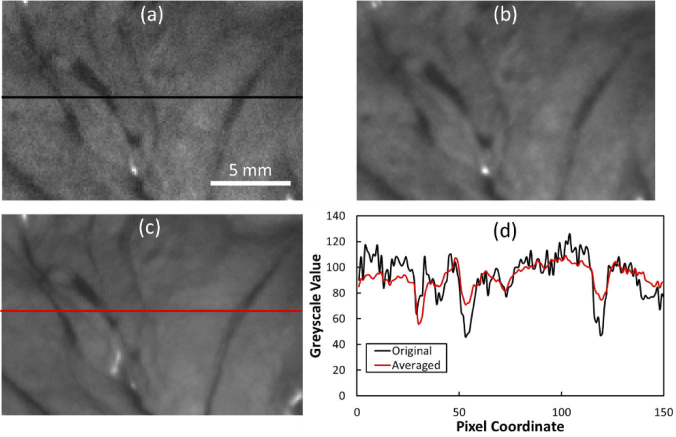
Temporal averaging. (a) Raw image of bowel serosa at 520 nm acquired using 33 ms exposure time and 24 dB gain. (b) Temporally-averaged image using six sequentially-acquired 520 nm images, without motion correction. (c) Temporal averaging of the same stack after image registration. (d) Greyscale profiles at the lines indicated in (a) and (c).

Due to high light absorption by tissue, at blue and green wavelengths in particular, and the relatively short integration times needed to achieve high frame rates, an appreciable level of noise was visible in some acquisition sequences (Fig. 4(a)). To improve the signal-to-noise ratio in these cases temporal averaging was performed using a short sequence of data cubes over ∼3-5 s. Motion artefacts due to respiration were non-negligible over this time scale, however, and resulting in blurring of fine spatial features (Fig. 4(b)). Therefore, a correction step was introduced using available image registration algorithms [25] implemented in Matlab’s Image Processing Toolbox (The Math Works, USA) to improve contrast with vascular features (Fig. 4(c)) and remove sharp, noisy, pixel intensity variations (Fig. 4(d)).

**Fig. 5.**
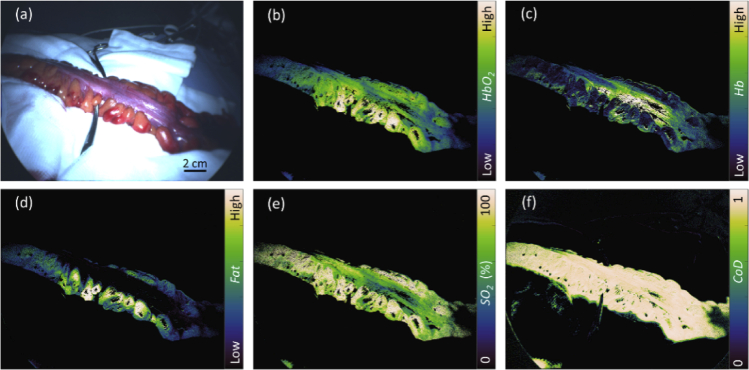
Processed images and concentration maps generated from the surgical MSI system data showing (a) colour image of bowel segment (proximal end to the left), (b) HbO_2_, (c) Hb, (d) Fat (e) SO_2_, (f) CoD. Concentration values for Hb, HbO_2_ and Fat are relative and vary with illumination conditions, therefore the maps here are indicated as ‘low’ or ‘high’. The forceps in (a) mark the position at which the surgeon plans to transect the bowel.

A fully-processed acquisition is shown in Fig. 5, where the colour image and the concentration maps were generated as described in Section 1. The serosal surface of the bowel is flanked by noticeably yellow adipose tissue attached to the mesentery, which corresponds to the bright regions shown on the *Fat* concentration map. The proximal end of the serosa is generally well oxygenated (∼75%) but this decreases distally, as seen in the *SO_2_* map. The transition between these two regions appears to correlate with the surgeon’s choice of transection point, indicated by the position of the forceps in Fig. 5(a). Oxygenation in the mesentery is not observed to follow this decrease.

**Fig. 6.**
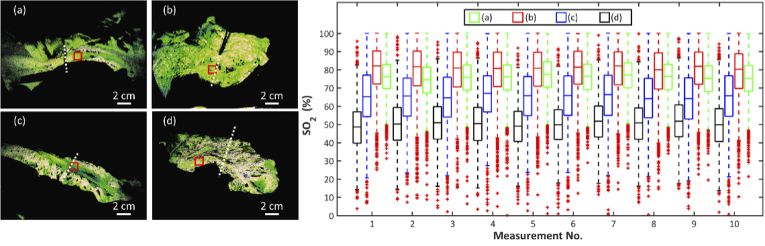
Temporal stability. Ten sequential acquisitions of the same organ in four cases with differing *SO_2_*. The boxplot shows the variation in *SO_2_* data within an ROI, indicated by the red boxes in each of the oxygenation maps (a)-(d). The location of the clinical decision is indicated by the white dashed line.

Reproducibility of the technique was assessed by imaging, using laparoscopic MSI, a segment of healthy bowel and acquiring image stacks continuously over a period of 3 s. The mean oxygen saturation in four regions-of-interest was calculated and plotted against measurement number (Fig. 6).

The measurement in each case is stable, with a variation (standard deviation) of approximately ±1% in the mean value. There is some low frequency variation in each of the four acquisitions shown, which is likely due to uncorrected respiratory motion artefacts. The high *SO_2_* regions shown in Fig. 6 (a and b) correspond to well-perfused portions of the colon, while the other regions, toward the distal end of the segments, have compromised blood supplies. These low *SO_2_* regions also lie in the part of the organ deemed by the surgeon to have sub-optimal perfusion (i.e., lying to the right of the forceps in Fig. 6(c) and (d)).

**Fig. 7.**
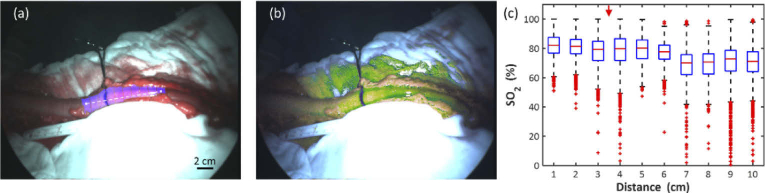
Oxygenation profile. (a) A line (dashed) was drawn along the bowel segment, marked at 1 cm intervals. Regions of interest (blue shaded areas) were defined perpendicularly to this line. The forceps and inked line indicate the surgeon’s choice of transection point. (b) *SO_2_* map showing spatial variation of oxygen saturation along the bowel. (c) Box plot showing *SO_2_* from each ROI plotted against region (proximal to distal). The red arrow at the top of the plot indicates the corresponding clinical decision.

Images of bowel segments were analysed by quantifying *SO_2_* along their length from proximal to distal, as shown in Fig. 7. This oxygenation profile was generated by first defining the coordinates of the start and end points of a line running parallel with the segment. This was then subdivided into ten divisions. ROIs were defined as rectangular masks aligned perpendicular to the profile line. A binary mask was created, using software employing a watershed segmentation technique [26], to ensure that only the visible exposed serosal surface was being analysed.

The *SO_2_* map in Fig. 7(b) shows that there is a high oxygen area at the proximal end, indicated by bright green/yellow pixels which falls off towards the distal end, indicated by darker green/blue pixels. In this case there appears to be a relatively sharp demarcation between these high and low *SO_2_* areas and this corresponds with the clinical decision. The numerical data in Fig. 7(c) show that in the well-perfused region mean *SO_2_* ranges from 78-82%, but this falls to 68-71% distally.

**Fig. 8.**
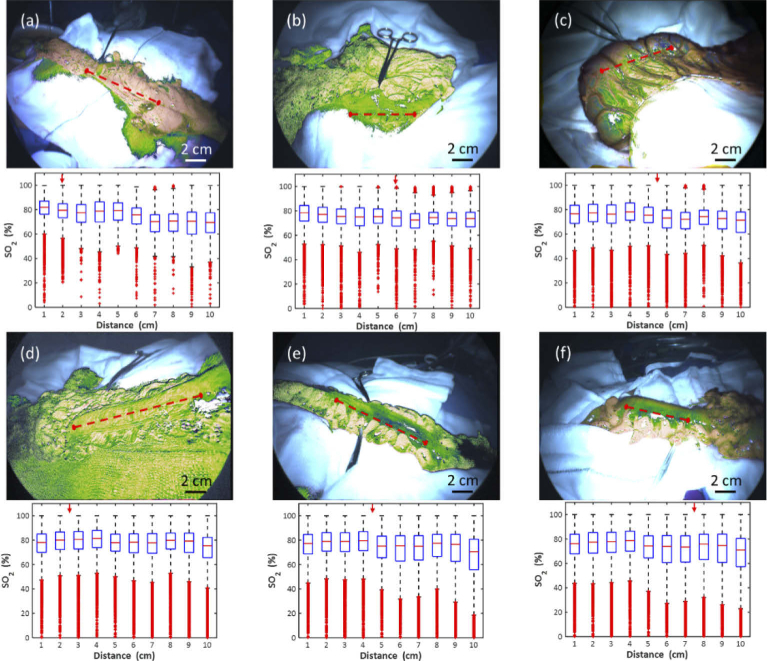
Oxygenation maps and corresponding profile box plots for six cases showing variation in *SO_2_* along the bowel segment. The clinical decision on transection is indicated by forceps in the image and a red arrow at the top of each plot.

When the same analysis is applied to a further six patients, as shown in Fig. 8 and Table 1, a similar downward trend from proximal to distal is observed in all cases (within the 50-90% range on the vertical axes). The observed decrease in median *SO_2_* is 7-12% in all cases, with the exception of Fig. 8(d), which represents the most subtle shift, with the first 4 cm having mean *SO_2_* of ∼80% and subsequent regions having mean *SO_2_* of ∼77%. There is also a trend for the *SO_2_* values in each ROI to become more varied, as can be seen from the interquartile range in the plots. The mean number of data points in each ROI was 6080 ± 1952. The clinical decision is seen to overlap with areas of relatively higher *SO_2_* in five of the six patients that exhibit a downward oxygenation trend, while one (Fig. 8(f)) appears to be in a region of relatively lower *SO_2_*.

**Table 1.** Tabulated *SO_2_* profile data plotted in Fig. 7 and Fig. 8, along with the corresponding position of the clinical decision for each patient.

	*SO_2_* (%)						
Position (cm)	P#1	P#2	P#3	P#4	P#5	P#6	P#7
1	82.14	81.91	78.37	76.50	78.11	77.33	76.21
2	81.54	79.56	77.06	77.41	80.05	79.08	77.52
3	79.38	77.45	75.45	76.37	80.65	79.02	77.89
4	79.82	78.92	75.01	78.25	81.38	79.57	78.64
5	80.30	79.33	75.33	75.47	77.95	75.12	74.27
6	77.64	75.76	74.37	73.10	78.38	75.43	73.93
7	70.20	70.18	72.49	72.08	77.80	75.08	73.42
8	70.72	70.80	74.52	74.41	79.81	77.56	75.83
9	72.84	70.61	73.63	72.69	79.39	76.58	74.76
10	71.14	69.50	73.61	71.39	75.45	70.61	71.04

Clinical decision (cm)	3.5	2	6	5.5	2.5	4.5	7.5

## 4. Discussion

The laparoscopic MSI system accurately measures spectral reflectance in the visible wavelength range, matching ground truth measurements from a high-resolution spectrometer to within 2%. Errors are marginally higher at the extreme ends of the spectrum, where the emitted light intensity of the xenon source is lower. During clinical measurements the speed of the filter wheel and camera meant that acquisition times for individual stacks were fast enough (∼300 ms) in comparison with breathing motion to not require any correction. However longer, time-averaged, sequences spanning several seconds can suffer from more appreciable alignment errors, which must be corrected using an image registration algorithm. Wavelength-dependent focus shifts were minimized through the use of the achromatic lens, while potential spectral distortions introduced by the filter wheel itself [27] were not observably significant in this set-up, as can be seen from the sharpness of the colour checker image in Fig. 3(a).

Previous point probe investigations of normal serosal oxygenation showed a high degree of inter-patient variability, with *SO_2_* values from 62-87% [3,4,28,29]. Furthermore, ligation of the inferior mesenteric artery (IMA) was seen to result in an absolute drop of almost 20% from baseline [28]. Similar results were observed in our study, with baseline measurements of mean serosal *SO_2_* in the range of 75-82%. A decrease in *SO_2_* was also noted in the imaging results, following IMA ligation, of ∼10% toward the distal end. In the cases documented in this paper the surgeon’s chosen transection point generally fell within the zone of higher oxygenation.

The mesentery was observed to have accumulated areas of adipose tissue, which were visibly yellow in comparison to the predominantly red surrounding mesentery and serosa. In these regions a linear model just including haemoglobin as an absorber fails to generate a good fit to the experimentally measured absorbance. Analysis of the residual signal shows a spectral shape strongly resembling that of adipose tissue. Inclusion of adipose tissue as an absorber significantly improves the quality of fit. Relative concentration maps of this absorber correspond strongly to the deposits visible in the colour images. Oxygen saturation in these high-fat regions of the mesentery is consistently higher than the serosa. Results from other groups also point to elevated perfusion in the mesentery [30] and an association between increased adipose tissue and higher *SO_2_* [31], although this has not been previously analysed in detail. While it is assumed that the adipose tissue observed in this study is exclusively white, MSI could potentially be used in other parts of the anatomy to quantify the contribution of brown adipose tissue and its impact on metabolic activity.

Most of the time taken for recording of MSI data was accounted for in positioning of the system at the bedside and removal once imaging was complete, with the actual imaging step taking just seconds. Therefore, there is potential for improvement with an optimised imaging system integrated in the operating theatre and used by the surgeons throughout the procedure. The effective frame rate of the system is close to 3 Hz, making it relatively robust to misalignment of the datacube, with only large motions, such as inspiration and expiration, causing significant errors. The processing algorithm described here is analytical and not optimized for speed. However, other work has demonstrated that alternative approaches, using machine learning are capable of reducing processing time to the millisecond range [32]. Therefore, in the context of being able to provide a snapshot of tissue perfusion intraoperatively, this system is capable of real-time operation. This compares favourably with competing perfusion assessment technologies, such as fluorescence image-guided surgery (FIGS), which require injection of a dye and temporal monitoring of the contrast signal.

The distance between tissue and the device was fixed during acquisition for every patient although there was variability between patients due to the constraints of the theatre environment. This means that the extracted absolute concentration values for haemoglobin and adipose tissue are not directly comparable. This effect may also have contributed to inter-patient variability.

A limitation of this experimental study is that no direct measurements of variables correlated with tissue perfusion were measured, like capillary lactates. It is plausible that there is interindividual variation not captured by the sample size of this study and, therefore, further cases are required. Furthermore, this approach should be prospectively tested, ideally in a randomized controlled trial setting, to more accurately quantify its potential benefit to patients. In such a trial imaging could also be performed on the completed anastomoses. Analysis of perianastomotic *SO_2_* in cases that subsequently leak could add further diagnostic value to the technique.

The MSI results used here could be used to support results from other clinical perfusion imaging modalities, such as FIGS. The link between a given fluorescence intensity and the underlying tissue properties is not clear and, despite investigation of quantitative analyses such as ‘time-to-peak’ [5], much of the routine use involves subjective interpretation of the fluorescence images.

## 5. Conclusions

The use of high throughput, high quality optical filters in the camera system described in this paper allows rapid acquisition of high spatial resolution images. The platform can deliver results intraoperatively, and relay these to the surgeon during the procedure. Further optimisation of the data processing pipeline would allow operation of a truly real-time system, integrated into the main imaging tools in the operating theatre, allowing the clinician to toggle rapidly between standard colour and oxygen saturation modes.

## Data Availability

Data will be made available on a UCL-hosted repository upon acceptance of the manuscript.
